# Genomics reveals historic and contemporary transmission dynamics of a bacterial disease among wildlife and livestock

**DOI:** 10.1038/ncomms11448

**Published:** 2016-05-11

**Authors:** Pauline L. Kamath, Jeffrey T. Foster, Kevin P. Drees, Gordon Luikart, Christine Quance, Neil J. Anderson, P. Ryan Clarke, Eric K. Cole, Mark L. Drew, William H. Edwards, Jack C. Rhyan, John J. Treanor, Rick L. Wallen, Patrick J. White, Suelee Robbe-Austerman, Paul C. Cross

**Affiliations:** 1 U.S. Geological Survey, Northern Rocky Mountain Science Center, Bozeman, Montana 59715, USA; 2 Center for Microbial Genetics and Genomics, Northern Arizona University, Flagstaff, Arizona 86011, USA; 3 Flathead Lake Biological Station, Division of Biological Sciences, University of Montana, Missoula, Montana 59812, USA; 4 USDA-APHIS, National Veterinary Services Laboratories, Ames, Iowa 50010, USA; 5 Montana Fish Wildlife and Parks, Bozeman, Montana 59718, USA; 6 USDA-APHIS, Veterinary Services, Fort Collins, Colorado 80526, USA; 7 USFWS, National Elk Refuge, Jackson, Wyoming 83001, USA; 8 Wildlife Health Laboratory, Idaho Department of Fish and Game, Caldwell, Idaho 83607, USA; 9 Wyoming Game and Fish Department, Laramie, Wyoming 82070, USA; 10 National Park Service, Yellowstone National Park, Mammoth, Wyoming 82190, USA

## Abstract

Whole-genome sequencing has provided fundamental insights into infectious disease epidemiology, but has rarely been used for examining transmission dynamics of a bacterial pathogen in wildlife. In the Greater Yellowstone Ecosystem (GYE), outbreaks of brucellosis have increased in cattle along with rising seroprevalence in elk. Here we use a genomic approach to examine *Brucella abortus* evolution, cross-species transmission and spatial spread in the GYE. We find that brucellosis was introduced into wildlife in this region at least five times. The diffusion rate varies among *Brucella* lineages (∼3 to 8 km per year) and over time. We also estimate 12 host transitions from bison to elk, and 5 from elk to bison. Our results support the notion that free-ranging elk are currently a self-sustaining brucellosis reservoir and the source of livestock infections, and that control measures in bison are unlikely to affect the dynamics of unrelated strains circulating in nearby elk populations.

## Abstract (web summary)

The role of wild elk in the spread and persistence of bovine brucellosis in the Great Yellowstone area is unclear. Here, Kamath *et al.* analyse the genomic sequences of 245 *Brucella abortus* isolates from elk, bison and cattle, supporting the idea that elk is an important reservoir and source of livestock infections.

## Graphical abstract

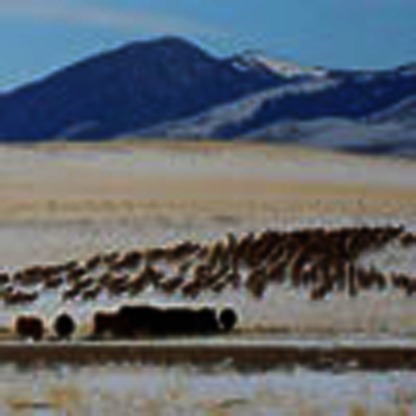

Whole-genome sequencing (WGS) can provide increased discriminatory power over traditional molecular typing approaches for inferring population dynamics and reconstructing transmission pathways of slowly evolving bacterial pathogens^1^,^2^,^3^. High-resolution genomic data have yielded novel insights into pathogen evolution^4^, outbreak dynamics^1^,^2^, cross-species transmission^5^,^6^ and geographic spread at both local^7^ and global^1^,^8^ spatial scales. While many studies have applied genomic approaches to better understand infectious disease epidemiology in humans and domestic animals, those examining bacterial disease dynamics in wildlife are lacking (but see refs 4, 6), in part due to the challenges of achieving sufficient high-density sampling of pathogen isolates from wild host populations.

Bayesian phylodynamic approaches that link the evolution and demography of a pathogen under a single-population dynamic framework have enabled investigations of infectious disease dynamics on timescales relevant to epidemiological and ecological processes^9^. Phylodynamic inference is dependent on the ability to detect evolutionary change in real time (over the time span of sampling)^10^ and, thus, its application has been extended to bacteria with advancements in WGS technologies^3^. Reconstruction of the past population history underlying a pathogen genealogy allows for the estimation of parameters, such as the rate of evolution^11^ and spatial spread^12^,^13^. Further incorporation of geographic or host data can elucidate outbreak dynamics and enable identification of infection sources and introduction dates into new hosts and locations^14^.

The identification of pathogen reservoirs is particularly challenging when pathogens infect multiple hosts, with some of the clearest evidence coming from the rare implementation of major control actions^15^. Ancestral state reconstruction of pathogen genealogies has emerged as a useful statistical approach to address this challenge. This method involves predicting branch and node states (for example, species and location of sampling) back through time based on the associated states of the collected samples^16^. Such an approach provided evidence that human *Salmonella typhimurium* cases in Scotland did not originate from local animal populations^5^. Similarly, the integration of viral sequences and host species data revealed patterns of rabies emergence, host shifts and cross-species transmission among bats in North America^17^,^18^.

Bovine brucellosis, caused by the bacterium *Brucella abortus*, is a zoonotic disease producing chronic infections in livestock, wildlife and humans worldwide^19^. Infections can lead to reproductive failures in female ungulates, with transmission primarily occurring through direct contact with aborted fetuses, placentas and birthing fluids^20^. Disease outbreaks can result in considerable financial losses for ranchers and the livestock industry. Therefore, brucellosis in cattle has been the focus of an aggressive eradication campaign over the last half century in the United States^21^. Today, the Greater Yellowstone Ecosystem (GYE), an expansive region crossing Idaho, Montana and Wyoming, is the last remaining reservoir of *B. abortus* within the country.

The source of contemporary brucellosis outbreaks in livestock within the GYE has been the subject of a contentious debate, particularly in recent years as the number of livestock cases has increased substantially across the tri-state region^22^. *B. abortus* was likely introduced into the GYE with infected cattle before 1917, when it was first detected in Yellowstone National Park (NP)^23^,^24^. Today, brucellosis continues to persist in wild bison (*Bison bison*) and elk (*Cervus canadensis*) populations, with occasional transmission to domestic bison and cattle. Formerly, bison were thought to be the primary wildlife reservoir, playing a more significant role than elk in transmission to cattle^25^,^26^. This belief emanated from serology data showing high levels of *B. abortus* exposure (that is, seroprevalence) in bison (approximately 40–60%) from Yellowstone NP, Grand Teton NP and the National Elk Refuge (NER)^26^,^27^,^28^,^29^,^30^,^31^. However, bison rarely move outside of conservation areas and are subject to rigorous management practices (for example, hazing, culling and hunting) that limit migration, dispersal and commingling with cattle^32^. In contrast, elk populations generally have lower levels of *B. abortus* seroprevalence (approximately 0–30%) throughout the GYE^26^,^33^. Elk are more numerous and widespread, have greater overlap with the distribution of livestock, and often make long-distance migrations between summering and wintering grounds^34^,^35^, thereby increasing the likelihood of contact with livestock.

Winter supplemental feeding of elk occurs at 23 sites in western Wyoming to reduce the loss of food stored for livestock and commingling of elk with cattle during the brucellosis transmission period^36^. *B. abortus* seroprevalence in elk using these high-density feeding areas has historically been elevated (10–30%) relative to populations using native wintering areas (2–3%) (refs 29, 33). More recently, however, *B. abortus* seroprevalence has been on the rise in some elk populations wintering on native ranges, in association with changes in population size and density^29^,^33^,^37^,^38^,^39^. In these populations, *B. abortus* exposure is comparable to elk using feedgrounds and expanding into new regions, which suggests elk may be a developing reservoir for brucellosis in the absence of feedgrounds and bison^33^,^39^. Genetic studies have also demonstrated elk are the most likely source of infections in livestock^22^,^40^,^41^. However, the role of elk in spatial spread and persistence in the GYE remains equivocal.

In this study, we evaluate the spatial and temporal dynamics of brucellosis transmission among wildlife and livestock in the GYE. Because the *Brucella* genus is highly conserved and has low genetic variation^42^, we use a WGS approach to create highly resolved time-calibrated phylogenies. We generate a robust genomic data set from *B. abortus* isolates collected from the three host species (cattle, bison and elk) over a 30-year period in the GYE, and apply phylodynamic methods that integrate pathogen genomic data with temporal, spatial and host phenotypic data. Our objectives are to (i) investigate the history and evolution of *B. abortus* in the GYE, (ii) identify *B. abortus* lineages associated with hosts and/or geographic localities and (iii) quantify brucellosis transmission across host species and populations. We present evidence for five genetically distinct *B. abortus* lineages in GYE wildlife with variable distributions and rates of spread. While elk-associated lineages historically derive from the feedgrounds, *B. abortus* is presently being self-sustained in elk independent of the feedgrounds or bison populations. This study demonstrates the value of WGS for examining bacterial pathogen transmission at a wildlife–livestock interface.

## Results

### *B. abortus* evolution and introduction history in the GYE

**Figure 1:**
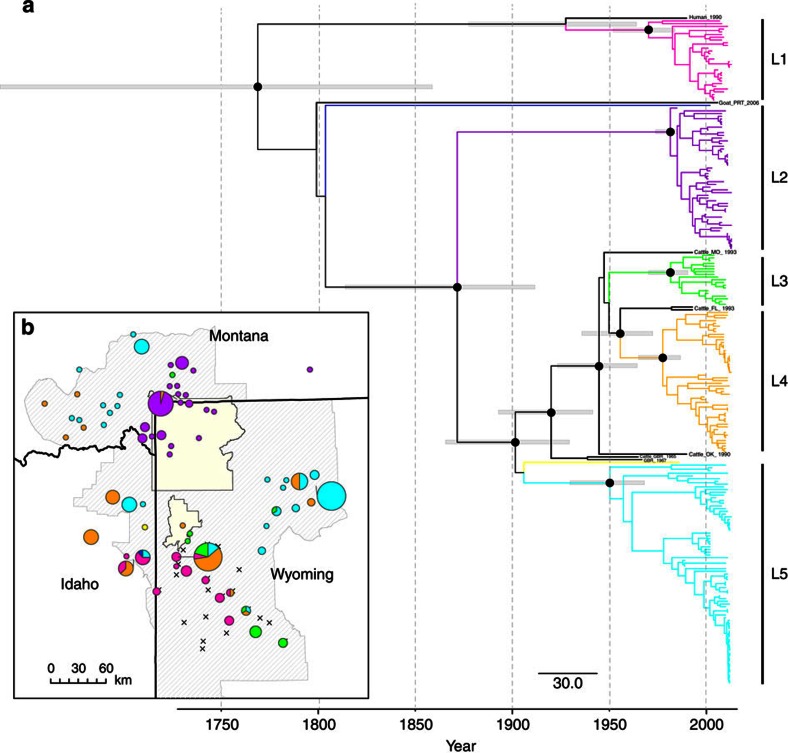
Time-calibrated maximum clade credibility tree and distribution of *B. abortus* lineages in the GYE. (**a**) Five major *B. abortus* lineages (L1–L5) identified through Bayesian phylogenetic analyses. Outgroups from outside the GYE (black), a vaccine-derived elk isolate (dark blue) and a single GYE cattle isolate (yellow) fell outside the major clades. Well-supported ancestral nodes (PP>0.95) are indicated by black circles (support for more recent nodes within major lineages are not shown) with grey bars indicating the 95% HPD interval for node date estimates. (**b**) Spatial distributions of *B. abortus* lineages are shown, with colours corresponding to the phylogeny. Pie charts represent the proportion of a particular lineage in a given location. Diagonal line shading represents the area where elk populations chronically infected with *Brucella* bacteria could potentially transmit brucellosis to livestock. The 23 elk feedgrounds (×) and national parks (pale yellow) are indicated.

WGS of the 245 *B. abortus* isolates from multiple hosts (Supplementary Table 1, Supplementary Fig. 1, Supplementary Data 1) enabled the identification of 1,463 single-nucleotide polymorphisms (SNPs; Supplementary Data 2). The time-measured *B. abortus* phylogeny, constructed under the best-fit relaxed lognormal clock and Bayesian skyline demographic models (Supplementary Table 2) indicated five divergent lineages (L1–L5; Fig. 1a), which suggests a minimum of five introductions of *B. abortus* into the GYE. Lineages shared no or few mutations (Supplementary Tables 3 and 4). Two GYE isolates fell outside these major clades: The first was derived from a vaccinated elk and may represent a vaccine strain, whereas the second was a 1986 cattle isolate, which possibly represents an introduction that was never transmitted to or established in wildlife. The evolutionary rate varied among branches, with the median rate (weighted by branch length) estimated as 1.4 × 10^−7^ substitutions per site per year, which corresponds to 0.46 SNPs per year per genome. However, branch-rate variation was low (95% highest probability density, HPD: 1.1–1.7 × 10^−7^ substitutions per site per year). The predicted date of the ancestral root of the tree was approximately 1769 (median=244 years before 2013) and this estimate was also the time to most recent common ancestor (tMRCA) for all GYE isolates. Estimates for tMRCA and clock rate were confirmed with the majority of clock and demographic model combinations, with the exception of the Bayesian skyride demographic and relaxed clock model prior, which received little support (Supplementary Tables 2 and 5). Within each primary lineage, the tMRCA date ranged from 1950 to 1982 (Fig. 1a, Table 1). Results from independent analyses by lineage corroborated these results, yielding similar tMRCAs for each lineage (Table 2). For the majority of cattle outbreaks, the median tMRCA was ≤2 years (range, 0–12 years) prior to the first detected brucellosis case in the herd (Supplementary Table 6, Supplementary Fig. 2).

### *B. abortus* cross-species transmission dynamics

**Figure 2:**
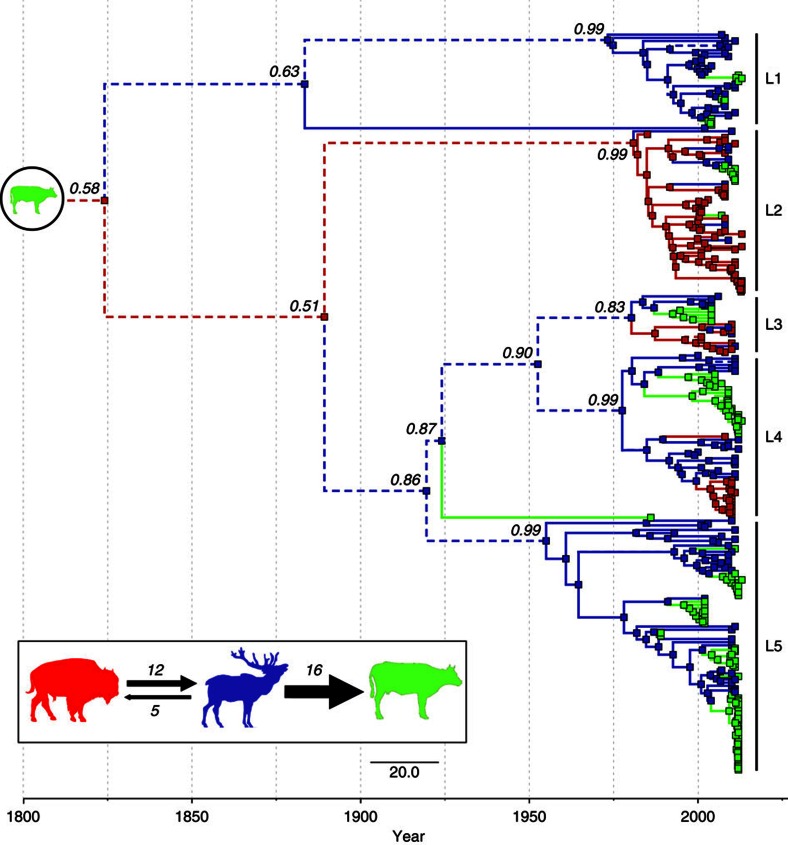
Ancestral host-state reconstruction over the *B. abortus* phylogeny. Maximum clade credibility tree under a model of asymmetric host species transitions. Host-state posterior probabilities (PP) are reported for ancestral nodes up to the MRCA for each lineage (L1–L5). Branches and nodes (squares) are annotated with their most probable (PP>0.5) host species states using colour labels (red=bison; blue=elk; green=livestock (that is, cattle and domestic bison)). Branches shown with dashed lines represent states that cannot be predicted accurately with the data used here given that the identified lineages likely represent separate introductions. Cattle were the original source of introduction into the GYE, and thus, are the hypothesized MRCA host state for all isolates (shown in circle at root).

Ancestral host-state reconstruction predicted wildlife as the most probable ancestral source for the current *B. abortus* lineages (Fig. 2). Specifically, elk was the highest probable ancestral host state for most lineages (L1, L4 and L5: posterior probability, PP=0.99, L3: PP=0.83), with the exception of L2, for which wild bison was predicted as the ancestral host (PP=0.99). According to reconstructions, the historical source of all GYE isolates was also bison, however, the host-state probability was low (PP=0.58). Host-state inference of the oldest nodes generally should be taken with caution because this analysis lacks representative isolates from cattle infections outside the GYE; therefore, we note that these results are expected to reflect the first wildlife species infected by cattle in the region.

Multiple (two–three) host species were observed within all major clades, with wildlife and livestock interspersed throughout the tree, supporting historical and continual cross-species transmission in the GYE (Fig. 2). However, the pattern of isolate clustering by host species (at branch tips) and the low proportion of host switches (∼15% of nodes; Table 3) suggest that the majority of transmission events have likely occurred within species. We also observed clustering at the herd-level for livestock where data on herd origin existed. Livestock herd outbreaks were generally independent and nested within elk clades (Fig. 2), with one exception of two genetically linked cattle outbreaks in Idaho (Supplementary Table 6). The estimated mean number of host transitions per year between each pair of species was low (range, 0.3–1.9 transitions per year) and only three of these rates (elk to livestock, bison to elk and elk to bison) were well supported (Bayes Factor, BF>100; Table 3). The number of host switches along the tree, based on a Markov jump counting procedure^43^, was highest from elk to livestock (median=16.4) and also substantial between wildlife species (bison to elk: 12.2, elk to bison: 5.2; Table 3). Analyses considering domestic bison and cattle as different host species yielded similar results (Supplementary Table 7).

### Spatial distribution and spread of *B. abortus* lineages

Three of the five major clades were geographically limited to Yellowstone NP and the adjacent Paradise Valley in Montana (L2), or the Wyoming feedgrounds (L1, L3; Fig. 1b). The other two lineages (L4, L5) exhibited a widespread distribution across the GYE. Phylogeographic diffusion analysis indicated *B. abortus* spatial dispersion was variable among branches. The posterior median rate of spatial spread was 2.9 km per year (95% HPD: 2.2–3.6 km per year) when including all isolates in a single analysis. This analysis predicted a common ancestor for all GYE brucellosis infections in the Jackson Hole and Grand Teton NP area, which encompasses the NER (Supplementary Fig. 3). There were multiple epidemiological linkages between this location and other areas, suggesting the area may be linked to long-distance brucellosis dispersal through time. Northward (to Yellowstone NP and Montana) and westward (to Idaho) spread appeared to be older and less frequent, with subsequent transmission occurring locally. In contrast, pathogen movement within Wyoming to the east and southeast of the feedgrounds was more frequent and recent (post-1990).

**Figure 3:**
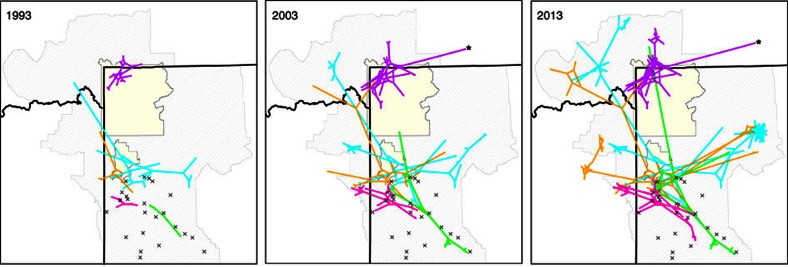
Spatiotemporal diffusion of *B. abortus* in the GYE. The spatial dispersion of *B. abortus* is shown at three time points (1993, 2003 and 2013), with coloured branches representing spatial projection of the *B. abortus* phylogeny on the first day of the calendar year. Five lineages are indicated by colours corresponding to Fig. 1. Feedgrounds (×) and conservation areas (pale yellow) are shown. Diagonal line shading represents the area where elk populations chronically infected with *Brucella* bacteria could potentially transmit brucellosis to livestock. Star indicates a documented long-distance cattle movement.

**Figure 4:**
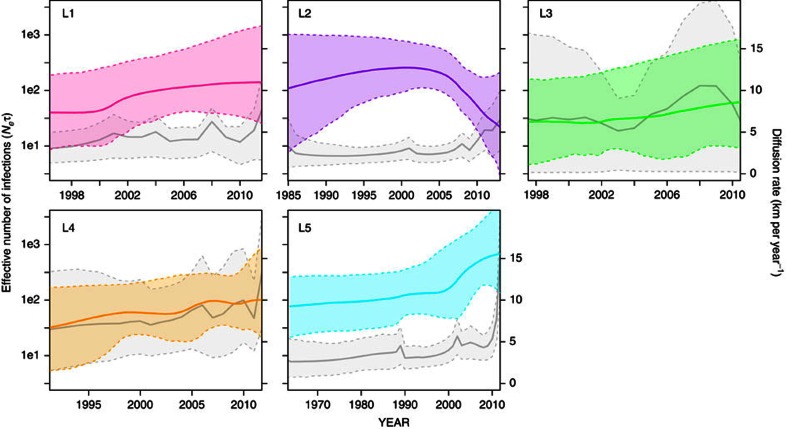
*Brucella* lineage-specific Bayesian skyline plots and diffusion rates over time. Skyline plots show the effective number of infections (*N*_e_*τ*) over time. Lines represent the median parameter estimates, with lineages (L1–L5) colour coded as in Fig. 1 and dashed lines representing the 95% HPD interval. For each lineage, the median diffusion rate over time is shown (solid grey line), with dashed lines representing the 80% HPD interval. Range of *x* axis differs for each lineage, starting at the lower 95% HPD limit of the root height estimate.

Spatial spread analyses by lineage, which more accurately assumes lineages represent separate introductions, suggested a Wyoming feedground origin for 4 out of 5 lineages (Fig. 3). The exception was for L2, which was predicted to originate in Yellowstone. L3 and L4 were evolving marginally faster than the other lineages (Table 2). Parameter estimates for L3, however, were generally imprecise with large 95% HPD intervals due to small sample size. Lineage 2 (predominantly Yellowstone bison) was evolving two to three times more slowly than the other lineages. Lineage-partitioned estimates of the tMRCA (Table 2) generally agreed with estimates from analyses including all the data. Dispersion rates were faster in recent time (that is, within lineages), with dispersion rate heterogeneity by lineage (range, 2.6–7.9 km per year; Table 2). Lineage 2 was dispersing the slowest (2.6 km per year) relative to other lineages. In contrast, L4 was spreading at a considerably faster rate (7.9 km per year). When only considering the recent GYE lineage data, the median diffusion rate was higher (4.2 km per year) than when including all samples in a single analysis (2.9 km per year). Lineage-specific rates of spatial spread also varied through time, increasing for most lineages since their estimated time of introduction (Fig. 4).

### Effective number of *B. abortus* infections over time

Bayesian skyline plots revealed recent increases in the effective number of infections in four out of five lineages, with particularly steep increases in L5 since 2000 (Fig. 4). The exception to this was the bison-dominated lineage, L2, for which effective infections have recently declined.

## Discussion

In this study, we found genomic evidence for at least five independent introductions of *B. abortus* into wildlife populations of the GYE. Our data suggest that brucellosis is currently persisting in some free-ranging elk populations outside of the feedgrounds. Most *Brucella* strains from native winter-range elk were genetically distinct from those infecting YNP bison and instead were historically connected to the supplemental feedgrounds in Wyoming. These elk-associated lineages have been spatially expanding at approximately 4–8 km per year. In contrast, bison from Yellowstone NP had predominantly one lineage of *B. abortus*, and this lineage had the slowest spatial diffusion rates of all five lineages. Furthermore, host-state reconstruction confirmed previous findings^22^,^40^,^41^ that elk were the most likely source of *B. abortus* outbreaks in livestock. This study is one of the few genomic studies examining bacterial transmission in a wildlife setting and highlights the important role phylodynamic approaches can play in understanding wildlife disease systems, even those involving slowly evolving pathogens.

Overall, the topology of the *B. abortus* phylogeny indicated a fair amount of cross-species transmission over evolutionary time, with the presence of multiple species interspersed within lineages. Even so, the clustering of isolates by host species in recent time implies the majority of transmission events are occurring within species. Using ancestral state reconstruction, we quantified the proportion of cross-species to within-species node transitions (as a proxy for relative transmission) and showed that only about 15% of phylogenetic nodes displayed a predicted host switch (Table 3).

Our data support the hypothesis that elk in the GYE are currently a significant source of brucellosis infections in livestock, adding to the accumulating evidence from recent ecological and genetic-based studies^22^,^33^,^40^,^41^. Over the past decade, in particular, there has been a substantial increase in documented transmission events from elk to livestock (*n*=17), standing in contrast to none recorded in the previous decade^22^. We also demonstrated that the quantity of predicted elk to livestock transitions (that is, Markov jumps over *B. abortus* evolution in the GYE) was greater than between any other host pair (Fig. 2, Table 3). In contrast, the predicted number of bison to livestock transitions was close to zero and no transmissions of brucellosis from wild bison to cattle have been detected. These results are not surprising given elk are more numerous and widely distributed than bison, which may increase their probability of contact with livestock.

These data also reveal significant transmission between bison and elk in areas of sympatry, suggesting that eradication efforts in one host population may be complicated by the probable reinfection from the alternative host species. Cross-species transmission was asymmetric with more than twice as many phylogenetic transitions from bison to elk than the reverse scenario. This asymmetry is perhaps expected given that *B. abortus* seroprevalence is typically much higher in bison than in elk^26^,^27^,^28^,^29^,^30^,^33^, which may in part be explained by differences in population densities among hosts and/or *B. abortus* tropism for bovines^44^. Transmission from elk to bison could be driven by management given that bidirectional host transitions were only observed in the NER where both species are fed in close proximity during the winter; whereas, host transitions were primarily unidirectional (from bison to elk) in Yellowstone NP where both species are free ranging and not artificially concentrated by supplemental feeding. However, because sympatric populations of bison and elk only occur within and near the national parks and the NER, any possible role for bison as a reservoir may only be relevant at small geographic scales. Thus, management actions (for example, vaccination, culling) directed towards bison in Yellowstone NP may not affect brucellosis prevalence elsewhere in the GYE. Also, *B. abortus* appears to be persisting in elk outside of these locations, so under present conditions it is unlikely that bison are a necessary host for brucellosis persistence.

Evolutionary rate predictions based on heterochronically sampled genetic data provide a unique opportunity to estimate the timing of disease introductions or emergence. The MRCA of all GYE isolates likely existed in the late 1700s (95% HPD: 1638–1862, Table 1). This ancestral root is probably more representative of the arrival of *B. abortus* to North America with European cattle than its introduction into the GYE. However, the arrival of *B. abortus* to North America could be much older than the MRCA due to the extinction of early lineages. In addition, the ages of the oldest divergence events may be underestimated if the total sampling interval of the study was too short (that is, sparse historical sampling), thus biasing the evolutionary rate prediction high^45^. Even though considerable genetic information was obtained in this study, the short sampling time frame relative to the depth of the tree may remain problematic for historical inferences in this and other disease investigations for which sampling may not be feasible across the entire time span of the phylogeny. If they can be found, then historical isolates and/or genomes of *B. abortus* from North America would likely improve these estimates. Despite this, we can conclude that our predicted MRCA likely predates the introduction of *B. abortus* into the GYE given that all outgroup isolates also share this ancestor.

The *B. abortus* genealogical structure provided evidence for multiple introductions (with 5 lineages) of brucellosis into the GYE (Fig. 1a). In 4 of 5 lineages, the MRCAs existed within a short time span (1970–1982), even though brucellosis was documented in the GYE as far back as 1917 (ref. 24). These earlier lineages may not have persisted or were present at low frequencies when isolates were sampled. Lineage disappearance may have been the result of stochastic extinction at low wildlife population sizes^46^. The three GYE States were not brucellosis free in their respective livestock populations until the mid to late 1980s, and extensive testing of cattle that entered the area was not routine. Therefore, multiple livestock-related introductions before and including the 1980s with spillover into elk wintering on cattle ranches is plausible, particularly since transmission has been observed in the reverse direction (from elk to cattle) 17 times between 2002 and 2012 (ref. 22).

Contemporaneous lineage MRCAs may alternatively reflect a time period of brucellosis expansion following a bottleneck because of lower bacterial prevalence and/or host population sizes. The tMRCAs for these lineages align with the cessation of large-scale management removals of bison and elk in the late 1960s, after which ecological processes such as competition and predation were allowed to prevail in national parks and wilderness areas with minimal human intervention. Under this new management paradigm, bison and elk numbers in Yellowstone NP increased rapidly from the 1970s through 1990s (refs 26, 28). Bison abundance rose from about 400 to 3,500 individuals, and elk abundance from 3,000 to 18,000 in Yellowstone from 1968 to 1990. Furthermore, in the late 1960s, a brucellosis-free bison herd was reintroduced in the Jackson Hole Wildlife Park. However, in 1975 this herd began spending winters on the nearby NER with infected elk and, by the late 1980s, 76% of culled bison tested positive for *B. abortus*^31^. Similarly, in Montana, some elk populations in the Madison and Paradise Valleys have increased five- to ninefold in abundance since 1975 (ref. 33). In contrast, the NER has supported large elk population sizes from the 1950s to present and, interestingly, this location also had the highest *B. abortus* lineage diversity. Therefore, we hypothesize that the predicted lineage MRCA dates may relate to a genetic bottleneck in *B. abortus* diversity due to lower wildlife host population sizes followed by geographic spread as elk populations increased in areas outside of the NER.

The time from brucellosis introduction to detection within livestock outbreaks was generally short (≤2 years; Supplementary Table 6, Supplementary Fig. 2), assuming that the MRCA represents the first case in the herd. This finding implies transmission opportunities among herds are limited, likely due to the strict quarantine and testing procedures required following a brucellosis diagnosis. This was corroborated by data showing the majority of domestic bison and cattle outbreaks were independent and nested within elk lineages, further supporting that livestock infections are primarily the result of spillover from elk. There was only one exception, where two cattle outbreaks in Idaho appeared to be genetically linked (Supplementary Table 6). Linked cattle herds could imply direct cattle-to-cattle transmission, or alternatively, that both herds were infected from the same unsampled elk source. In a few cases the time to detection was surprisingly longer (>5 years) than would be expected under the brucellosis surveillance regime. However, testing was only required for cattle being sent to slaughter and, thus, a delay in detection may be plausible under the situation where infection prevalence was low and only a small proportion of the herd was slaughtered.

Spatial structure among *B. abortus* lineages varied, with two widespread lineages and three lineages limited in geographic distribution (Figs 1b and 3). The lack of spatial expansion in some lineages may be explained by time, limited host movements and/or low transmissibility as a result of genetic differences among strains. Most notably, *B. abortus* cases within Yellowstone NP and directly north of the park in Montana (that is, L2) appeared to be genetically distinct, having little spatial overlap with the other lineages. This implies that pathogen dispersal in or out of this area is likely rare, particularly in recent time.

Spatial spread analyses by lineage identified the Wyoming feedgrounds as an ancestral source for the majority of GYE isolates sampled within the past three decades (Fig. 3, Supplementary Fig. 3). The most widespread lineages (L4, L5), in particular, originated from the NER and exhibited multiple long-distance linkages with other areas in the GYE. Interestingly, the most probable dates of the oldest lineage MRCA (L5, 95% HPD: 1930–1968) immediately follows the first diagnosis of brucellosis in NER elk in 1930 (ref. 47). The bison-dominated lineage, however, was predicted to originate within Yellowstone NP, and may represent the remnants of the first reported introduction of brucellosis into the GYE in the early 1900s (ref. 23).

The overall rate of *Brucella* dispersal in the GYE was relatively low (∼3 km per year). This estimate was comparable to the overall phylogenetic dispersal rate of the bacterium *Mycobacterium bovis* in cattle of Northern Ireland, which was estimated at ∼2 km per year (ref. 7). Similar to *M. bovis*, we observed heterogeneity in diffusion rates among *Brucella* lineages (3–8 km per year; Table 2), and dispersal rates increased in recent time within most lineages (Fig. 4). Elk are capable of long-distance migratory movements and are the likely determinants of *B. abortus* spread throughout the GYE. Some of this spatial spread may be due to increased sampling effort in elk populations farther away from Yellowstone NP or the NER. We believe, however, that the sampling of isolates is itself driven by the spatial spread of *Brucella*. Obviously, one cannot recover isolates from regions where the pathogen does not yet exist. If we assume a constant rate of future spread of 8 km per year, the fastest lineages could be detected in the Bitterroot Valley of Montana or northeastern Utah within ∼20 years, or even sooner given that dispersal rates could presently be much higher in the two widespread lineages (L4, L5: >10 km per year; Fig. 4).

Coalescent-based analyses of lineage demographic histories indicated that there has been an increase in the effective number of infected hosts within most *Brucella* lineages since their estimated time of introduction (Fig. 4). Lineage 5 exhibited a particularly rapid increase since around 2000. In contrast, the effective number of infections in L2 has decreased since 2005, a finding that is contradictory with the lack of a coincident decrease in the seroprevalence of YNP bison^48^. The rate of coalescence, however, has been shown to be more reflective of new transmissions (that is, incidence rate)^49^, and because incidence and prevalence are theoretically out of phase, we may expect to see a corresponding lagged response in prevalence.

We identified ∼1,200 SNPs in *B. abortus* of the GYE and the median evolutionary rate was 1.4 × 10^−7^ substitutions per site per year. While there is substantial variation in estimated molecular clock rates across bacterial species, this estimated rate is comparable to other bacteria for which WGS data are available (for example, *Salmonella*: 3.4 × 10^−7^ substitutions per site per yr; ref. 5). A more realistic relaxed clock model best fit our data, indicating heterogeneity in branch-specific evolutionary rates. However, lineage-specific analyses suggested the overall variation is likely low (range, 0.7–2.2 × 10^−7^ substitutions per site per year). Variable rates of molecular evolution among bacterial lineages could be explained by natural selection, host demography or pathogen transmission history (for example, *Yersinia pestis*^8^). Thus, the observation that the bison-dominated lineage is evolving the slowest (Table 2) may in part be a result of host-specific strain adaptation, differences in the extent of spatial expansion, and/or the number of transmission bottlenecks.

Molecular techniques based on pathogen genetic data can provide valuable insights into disease transmission dynamics. However, these data are only capable of quantifying relative transmission at the population level and over evolutionary timescales, whereas specific epidemiological reconstructions of ‘who-infected-whom' cannot be determined unless nearly 100% of infections are sampled (but see ref. 50). Benavides *et al.*^51^ showed that phylogeny-based inferences regarding the quantity and directionality of transmission are sensitive to the total proportion of infections sampled and ability to obtain a balanced sample, respectively. Balanced sampling that is proportional to disease prevalence, however, is difficult to attain because the true prevalence within host populations is not known and sampling from wildlife is more difficult than from livestock. Despite this issue, we predicted contemporary livestock outbreaks in the GYE were the result of spillover from elk. Recent studies have presented some solutions for dealing with the problem of estimating infection probabilities and transmission from pathogen phylogenies in situations where there is incomplete sampling (for example, ref. 50). The implications of partial and unbalanced sampling to transmission inferences derived from genealogies remains an important focal area for future research.

This study demonstrates the value of WGS and phylodynamics for epidemiological inferences of bacterial pathogens at the wildlife/livestock interface. The additional resolution allowed us to identify multiple historical introduction events into wildlife and makes a compelling case for a new reservoir status of elk that is independent from bison in several regions. Integration of spatial and temporal information enabled the estimation of diffusion rates on a lineage-by-lineage basis, suggesting the fastest lineage is moving at ∼8 km per year. Future work connecting this spatial diffusion to habitat covariates, host movement and genetic connectivity, and serology may allow for predictive models that forecast not only the speed, but also the directionality of pathogen spread.

## Methods

### Sampling and isolate datasets

*B. abortus* isolates were obtained from naturally infected livestock (cattle, domestic bison) and wildlife (elk, bison) tissue samples collected over 48 years (1965–2013, *n*=245, Supplementary Table 1, Supplementary Data 1) using standard isolation protocols^52^. Briefly, each tissue was dipped in 95% ethanol and flamed, then homogenized with an equal volume of PBS. This suspension was then swabbed onto non-selective and selective agars including trypticase soy agar with 5% serum, trypticase soy agar with 5% serum and antibiotics, trypticase soy agar with 5% serum, antibiotics and ethyl-violet, Ewalt's media and Farrell's media with 5% serum. Agar plates were incubated at 37 °C and 10% CO_2_ for a minimum of 10 days, with observations typically at 5 and 10 days.

The majority of *B. abortus* isolates were derived from animals within the GYE (*n*=237) during 1985 to 2013 (Supplementary Table 1, Supplementary Fig. 1). We set the GYE isolates in a broader context by including eight additional samples from outside the region. Livestock samples were submitted to the National Veterinary Services Laboratories (Ames, IA, USA) through the National Brucellosis Eradication Program managed by the Animal and Plant Health Inspection Service of the U.S. Department of Agriculture (USDA). Wildlife-derived isolates were contributed by Federal and State wildlife agencies (see Acknowledgements). All samples were collected under the guidelines of the program as outlined in regulations 9CFR, Brucellosis Uniform Methods and Rules, and various APHIS Veterinary Services Memoranda.

### Whole-genome sequencing and SNP analysis

Total genomic DNA was extracted enzymatically from cultured *B. abortus* isolates using a commercial kit (MasterPure, Epicentre, Madison, WI, USA). Libraries for Illumina sequencing were prepared as previously described^53^. Briefly, genomic DNA was processed with a NexteraXT kit (Illumina, San Diego, CA, USA) according to the manufacturer's instructions. Alternatively, some samples were fragmented with a Matrical SonicMan microplate sonicator and processed with a KAPA Illumina Library Preparation Kit ‘With-bead' (KAPA Biosystems, Woburn, MA, USA). Paired-end libraries were sequenced with an Illumina GAIIx to produce 2 × 100 bp reads, or with an Illumina MiSeq to produce either 2 × 250 bp or 2 × 300 bp reads. SNP analysis was conducted using the NASP v. 0.9.1 pipeline (https://github.com/TGenNorth/NASP), using default settings for each analysis package implemented by the pipeline. Briefly, *B. abortus* genomes in FASTA format were aligned to the *Brucella abortus* 2308 reference genome (GenBank accession codes NC_007618, NC_007624) and analysed for SNPs with MUMmer 3.23 (ref. 54), whereas shotgun sequence reads were aligned with the Burrows-Wheeler Aligner 0.7.5a mem algorithm^55^ and SNPs called with the Genome Analysis Toolkit 2.5.2 Unified Genotyper^56^. Orthologous SNPs were selected for phylogenetic analysis with the following criteria. SNP loci were required to have a base call in all samples. SNPs in samples represented by sequence reads had a minimum of 10 × read coverage at a locus, and 90% of the base calls in agreement. SNP loci in duplicated regions, determined by an alignment of the reference to itself with MUMmer, were eliminated from further analysis.

### Phylogenetic reconstruction of *B. abortus* over time

We reconstructed evolutionary relationships among *B. abortus* isolates using a Bayesian coalescent Markov chain Monte Carlo (MCMC) analysis in BEAST v1.8 (ref. 57), integrating molecular sequence and temporal information on the sampling dates of the bacterial genomes to reconstruct a time-measured phylogeny. Data alignments included only SNPs to reduce computational time, but invariant sites (A: 700,366; C: 936,543; G: 938,280; T: 701,655) were accounted for by adjusting the pattern block to a total genome size of ∼3.28 Mbp. Because recombination can lead to erroneous estimations of phylogenetic relationships^58^, a Φ-test was conducted in SplitsTree^59^. Results were not significant (*P*=0.99) suggesting that recombination did not affect our interpretations.

We applied a General Time Reversible nucleotide substitution model with gamma-distributed rate variation (GTR+Γ) in phylogenetic analyses. We used a marginal likelihood estimation (MLE) model selection approach^60^ to determine the best-fit clock and demographic models. Two molecular clock models (strict and lognormal relaxed) were evaluated in combination with three coalescent demographic models: (1) constant population size^11^,^61^; (2) Bayesian skyline^62^; and (3) Gaussian Markov random field Bayesian skyride^63^. Model performance was evaluated by MLE based on path and stepping-stone sampling^60^ and paired comparisons of marginal likelihoods using BF^64^. The best-fit clock model prior was determined to be the uncorrelated lognormal relaxed clock model (Supplementary Table 2), which allowed for the evolutionary rate to vary among branches of the tree, with a continuous-time Markov chain^65^. The best tree model prior was the coalescent flexible Bayesian skyline model of demographic growth^62^, for which we specified 10 partitions with piecewise linear change.

Four independent MCMC analyses were each run for 100 million generations, utilizing the BEAGLE library^66^ to improve computational performance. Posterior distributions were sampled every 10,000 generations, and model parameters were assessed for convergence and satisfactory effective sample sizes (>200) in Tracer v1.6 (ref. 67). Trees were subsampled in LogCombiner and a maximum clade credibility tree was identified in TreeAnnotator^57^ after discarding the first 10% of trees as burn-in.

We estimated *B. abortus* evolutionary rates and MRCA dates for all samples and individual lineages. We defined a phylogenetic lineage as being the largest monophyletic cluster of individual GYE isolates that excludes outgroup samples and that is also highly supported (PP>0.95). The number of distinct lineages was assumed to reflect the minimum number of *B. abortus* introductions into the GYE; this operational definition assumes the GYE is not a source for recent (post-1985) infections outside of the system, which is reasonable due to the rigorous testing of exported animals. The number of fixed differences within and polymorphic differences among *Brucella* lineages was calculated in DNAsp v.5 (ref. 68).

### Bayesian estimation of host transition rates

We modelled host species as a discrete trait for each *B. abortus* lineage over the genealogy by ancestral state inference using a discrete phylogenetic diffusion model^69^ in BEAST v1.8 (ref. 57). This approach estimates the probability of the internal nodes and branches being associated with a specific host, based on relationships among host states of samples at the branch tips. Following ancestral state reconstruction over the genealogy, rate changes between discrete host states may be estimated over time by incorporating isolate sampling date. We considered three host species states: elk, bison and livestock (combining domestic bison and cattle into one group assuming a similar epidemiological role in the transmission process). We repeated these analyses considering four states (elk, bison, cattle and domestic bison) to account for the possibility of unknown species-specific transmission properties. Both analyses only included samples collected within the GYE (*N*=237; elk=85, bison=58, domestic bison=38 and cattle=56).

We assumed a diffusion model with asymmetric rates between pairs of host states after evaluating both symmetric and asymmetric models with MLE model selection^60^. A Bayesian stochastic search variable selection procedure^69^ was employed to allow for transitions between specific host pairs to be included or excluded from the model, and enable appraisal of the support for specific host transitions through BF calculations. Results from the multiple runs were combined, from which we identified well-supported (BF>10) non-zero host transition rates. We quantified the magnitude of host transitions using a robust Markov jump counting procedure that determines the posterior expectations of the number of host transitions along the branches of the tree^43^. For each host pair, the node transition proportion was quantified as the number of host species jumps divided by the total number of nodes in the tree (*n*=265). Four independent MCMC analyses were run for 100 million generations, sampling every 10,000, and combined. Convergence diagnostics were assessed. The quantified transition rates reflect relative rates of cross-species transmission, but do not enumerate true transmission rates because unobserved transmission events may occur along branches of the phylogeny.

### Lineage-specific evolution and spatial dispersion

We modelled the spatial dispersion of *B. abortus* GYE lineages over time and over a continuous landscape with a phylogeographic approach that accommodates branch-specific variation in dispersal rates^16^. This approach reconstructed two-dimensional character states in latitude/longitude coordinates of ancestral phylogenetic nodes. Using the MLE model selection procedure, we evaluated models that assume no branch-specific rate variation in dispersal rate (homogeneous Brownian diffusion) versus relaxed random walk (RRW) models (cauchy RRW, lognormal RRW and gamma RRW) that assume different distributions for rate variation among branches. The lognormal RRW best fit the data, and therefore, was applied to final analyses. We conducted and combined multiple MCMC runs (3 per lineage) of 100 million chains, sampled posterior distributions every 1,000 generations, subsampled to a total of ∼20,000 trees, and identified the maximum clade credibility tree after a 10% burn-in.

Analyses were run using all data and partitioned by lineage, to account for possible multiple introductions from sources outside the GYE. From lineage-partitioned models, we estimated lineage-specific evolutionary rates, tMRCAs, spatial dispersion rates and the effective number of infections as a product of the bacterial effective population size (*N*_e_) and generation time (*τ*) in years. The demographic history of each *Brucella* lineage (that is, change in *N*_e_τ over time) was evaluated using a coalescent-based flexible Bayesian skyline plot^62^, after pruning each livestock outbreak to only one isolate. We visualized spatial-temporal projections of *B. abortus* spatial spread in the GYE using the SPREAD program^70^.

## Additional information

**Accession codes:** Raw sequence reads have been deposited in the NCBI Sequence Read Archive (SRA) under BioProject accession PRJNA251693 (http://www.ncbi.nlm.nih.gov/bioproject/?term=PRJNA251693). Individual isolate accession codes are listed with associated metadata in Supplementary Data 1. Three previously published genomes were also obtained through the SRA (accession codes SRS401477, SRS401555 and SRS401602).

**How to cite this article:** Kamath, P. L. *et al.* Genomics reveals historic and contemporary transmission dynamics of a bacterial disease among wildlife and livestock. *Nat. Commun.* 7:11448 doi: 10.1038/ncomms11448 (2016).

## Supplementary Material

Supplementary Information Supplementary Figures 1-3 and Supplementary Tables 1-7

Supplementary Data 1 *Brucella abortus* isolate-associated metadata and accession codes.

Supplementary Data 2 *Brucella abortus* SNP matrix.

## Figures and Tables

**Table 1:** Posterior median estimates of genetic, temporal and spatial parameters.

**Parameter**	Median (95% HPD)*
Clock rate (× 10^−7^)†	1.40 (1.09–1.73)
Coefficient of variation‡	0.63 (0.46–0.84)
Root height date	1769 (1638–1862)
MRCA date (L1)	1970 (1952–1985)
MRCA date (L2)	1981 (1973–1985)
MRCA date (L3)	1982 (1970–1990)
MRCA date (L4)	1978 (1965–1987)
MRCA date (L5)	1950 (1930–1968)
Dispersion rate§	2.9 (2.2–3.6)

HPD, highest probability density; MRCA, Most recent common ancestor for GYE lineages (L1–L5).

^*^Estimated from a single analysis that included all isolates.

^†^Clock rate represents the Mean rate estimate weighted by branch lengths in units of substitutions per site per year.

^‡^Measure of the variation in evolutionary rate among branches.

^§^Dispersion rate in units of km per year, under model assuming dispersal rate varies among branches.

**Table 2:** Posterior median estimates of *Brucella abortus* lineage-specific model parameters.

**GYE lineage**	***n***	COV (95% HPD)*	**Clock rate (95% HPD)**†	**Diffusion rate (95% HPD)**‡	**tMRCA (95% HPD)**§	**Date**
L1 (pink)	30	0.13 (0.00–0.45)	1.64 (0.79–2.57)	3.5 (1.7–5.6)	34 (19–57)	1979
L2 (purple)	53	0.49 (0.09–0.89)	0.70 (0.43–0.96)	2.6 (1.6–3.6)	39 (28–60)	1974
L3 (green)	19	1.33 (0.69–2.13)	1.97 (0.07–3.87)	6.2 (0.2–12.3)	28 (13–118)	1985
L4 (orange)	52	0.97 (0.43–1.62)	2.15 (1.15–3.24)	7.9 (4.3–11.8)	31 (18–53)	1982
L5 (blue)	81	0.88 (0.40–1.46)	1.51 (1.05–2.00)	4.3 (3.0–5.7)	65 (40–105)	1948

COV, coefficient of variation; GYE, Greater Yellowstone Ecosystem; HPD, highest probability density; tMRCA, time to most recent common ancestor.

^*^COV: a measure of the variation in evolutionary rate among branches, 95% HPD interval.

^†^Mean rate estimate weighted by branch lengths in units of 10^−7^ substitutions per nucleotide per year.

^‡^Units in km per year.

^§^tMRCA reported in number of years prior to 2013.

**Table 3:** Model estimates for host-state transitioning between pairs of hosts.

**From**	**To**	Rate (95% HPD)*	**No. of Jumps (95% HPD)**†	**Jump proportion**‡	**BF**§
Elk	Bison	0.59 (0.02–1.42)	5.24 (2.05–7.71)	0.021	110
	Livestock	1.68 (0.25–3.46)	16.36 (14.23–19.16)	0.070	44,206
Bison	Elk	1.85 (0.27–3.79)	12.24 (7.26–18.50)	0.053	44,206
	Livestock	0.33 (0.00–2.32)	0.00 (0.00–1.56)	0.002	<3
Livestock	Bison	0.31 (0.00–2.30)	0.00 (0.00–0.29)	0.000	<3
	Elk	0.36 (0.00–1.96)	0.06 (0.00–2.41)	0.002	<3

BF, Bayes Factor; HPD, highest probability density.

^*^Rate represents the mean number of host transitions per year.

^†^Median estimate of the total number of Markov jumps over the phylogeny.

^‡^Proportion of transition nodes to total nodes in the phylogeny.

^§^BF support values.
