# Supplementary material for: Genomics reveals historic and contemporary transmission dynamics of a bacterial disease among wildlife and livestock
Source: Nat Commun. 2016 May 11;7:11448. doi: 10.1038/ncomms11448 (PMC4865865; doi:10.1038/ncomms11448)
Supplement: Supplementary Information — Supplementary Figures 1-3 and Supplementary Tables 1-7 [file ncomms11448-s1.pdf]

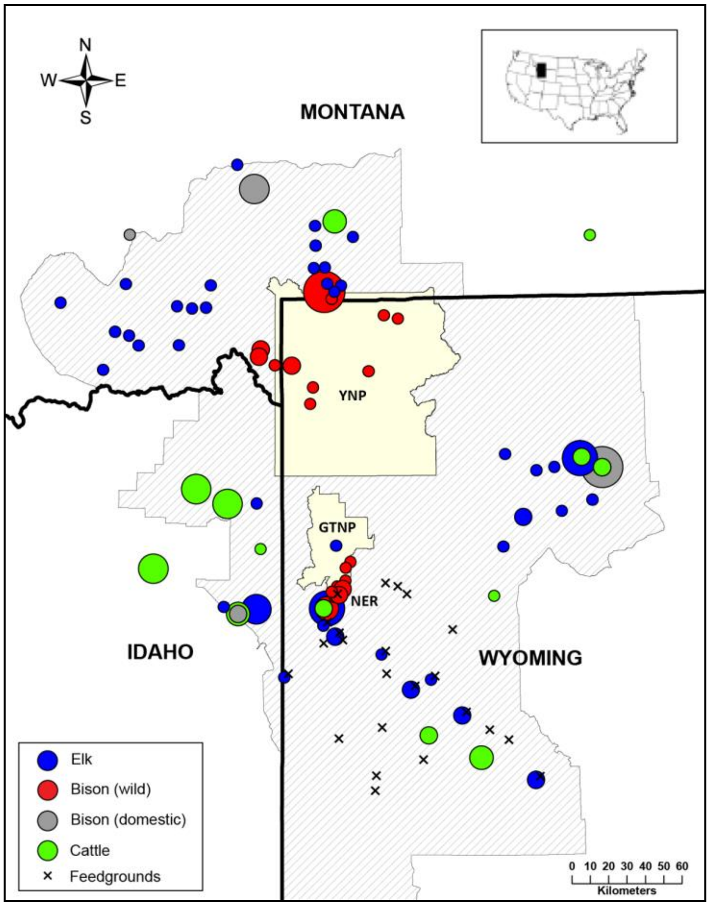

Supplementary Figure 1. *Brucella abortus* sampling by host species in the Greater Yellowstone Ecosystem. Sampling by location and host species reported with circles (elk = blue; wild bison = red; domestic bison = gray; cattle = green), with circle size relative to sample size (Range: 1 to 26). The 23 Wyoming elk feedgrounds are indicated (×). Livestock locations represent the last location prior to slaughter. Diagonal line shading represents the area in 2015 where elk populations chronically infected with *Brucella* bacteria could potentially transmit brucellosis to livestock. YNP = Yellowstone National Park; GTNP = Grand Teton National Park; NER = National Elk Refuge.

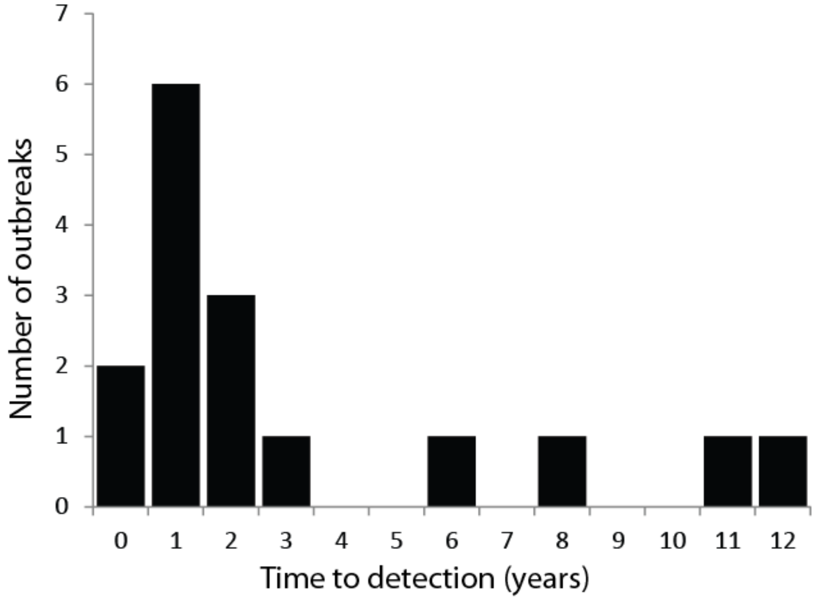

Supplementary Figure 2. Frequency distribution of brucellosis detection time within livestock herd outbreaks.

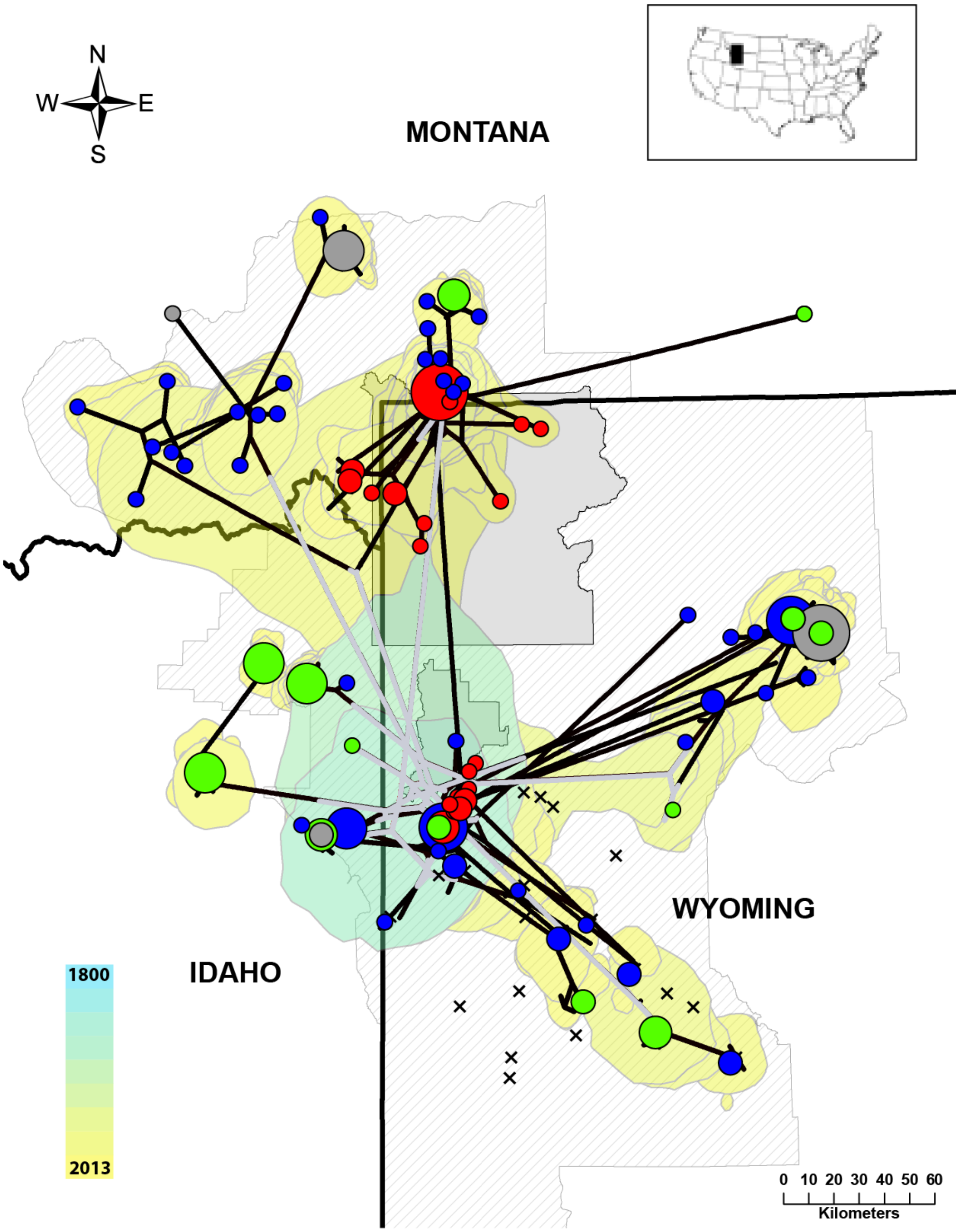

Supplementary Figure 3. Continuous phylogeographic diffusion model of *B. abortus* spatial spread in the GYE. This analysis includes all isolates and does not consider the different *Brucella* lineages as possible introductions. Polygons show the 80% highest probability density for the extent of spatial dispersion, with the relative time of dispersal represented by a blue to yellow gradient corresponding to the time period from approximately 1800 to 2013. The *B. abortus* time-measured phylogeny is overlaid on the map with historical (pre-1990) and contemporary (post-1990) dispersal branches represented in gray and black, respectively. Observed brucellosis cases shown at the tips by host species (elk = blue; wild bison = red; domestic bison = gray; cattle = green).

Supplementary Table 1. Sampling summary of *B. abortus* isolates.

	Host species	State or Country*	*N*	Sampling period
**Inside GYE**	*Elk*	ID	10	2001-2005
		MT	21	2005-2012
		WY	54	1993-2011
	Bison (wild)	MT	31	1985-2013
		WY	27	2000-2011
	Bison (domestic)	ID	3	2012-2013
		MT	9	2010-2013
		WY	26	2010-2012
	Cattle	ID	29	1986-2013
		MT	7	2007-2011
		WY	20	1989-2011
	**TOTAL**		**237**	
**Outside GYE**	*Cattle*	FL	2	1993
		MO	1	1993
		OK	1	1990
		GBR	1	1965
	Goat	PRT	1	2006
	Human	USA	1	1990
	Unknown	GBR	1	1967
	**TOTAL**		**8**	

*Abbreviations: ID (Idaho), MT (Montana), WY (Wyoming), FL (Florida), MO (Missouri), OK (Oklahoma), GBR (Great Britain), and PRT (Portugal).

Supplementary Table 2. Bayes factor values for MLE path sampling model selection of clock and demographic model combinations. Selected model is highlighted.

Clock Model		**Strict**			**Relaxed lognormal**		
	Demographic Model	*Constant*	*Skyline*	*Skyride*	*Constant*	*Skyline*	*Skyride*
**Strict**	*Constant*	---	-41.8	137.5	-34.3	-75.7	19.1
	Skyline	41.8	---	179.3	7.5	-33.9	60.9
	Skyride	-137.5	-179.3	---	-171.8	-213.2	-118.4
**Relaxed lognormal**	*Constant*	34.3	-7.5	171.8	---	-41.4	53.4
	Skyline	75.7	33.9	213.2	41.4	---	94.8
	Skyride	-19.1	-60.9	118.4	-53.4	-94.8	---

Stepping-stone sampling (not shown here) produced similar results

Supplementary Table 3. Number of fixed polymorphic differences (above diagonal) and shared mutations (below diagonal) between *Brucella* lineages in the Greater Yellowstone Ecosystem.

Lineage	**L1**	**L2**	**L3**	**L4**	**L5**
**L1**	---	186	177	177	177
**L2**	0	---	85	85	85
**L3**	0	0	---	26	52
**L4**	1	0	9	---	52
**L5**	0	0	7	10	---

Supplementary Table 4. The number of fixed differences and shared polymorphisms for each *Brucella* lineage compared with all other isolate sequences in the Greater Yellowstone Ecosystem. The number of sequences (*n*) and polymorphic sites (*P*) per lineage are shown.

Lineage	***n***	***P***	**Fixed differences**	**Shared mutations**
**L1**	30	146	139	1
**L2**	53	135	47	0
**L3**	19	99	13	10
**L4**	52	185	13	14
**L5**	81	293	26	11

Supplementary Table 5. Comparison and statistical performance of clock and demographic models.

Molecular clock model	Demographic model	Root height (95% HPD)*	Clock Rate (95% HPD) †	COV (95% HPD) ‡	Log marginal likelihood
**Strict**	*Constant*	256 (216-303)	1.28 (1.08-1.46)	n/a	-4533280
	Skyline	258 (215-311)	1.25 (1.06-1.44)	n/a	-4533239
	Skyride	163 (139-189)	1.59 (1.36-1.80)	n/a	-4533418
**Relaxed**	*Constant*	271 (171-408)	1.40 (1.11-1.70)	0.69 (0.51-0.90)	-4533246
	Skyline	244 (151-375)	1.40 (1.09-1.73)	0.63 (0.46-0.84)	-4533205
	Skyride	52 (48-58)	2.40 (2.07-2.77)	1.54 (1.15-1.96)	-4533299

n/a, not applicable

*Units are in number of years prior to 2013, HPD, highest probability density

†Units are 10^{-7} substitutions per nucleotide per year

‡COV: Coefficient of variation, which is a measure of the variation in evolutionary rate among branches

Supplementary Table 6. Estimated median time to brucellosis detection during livestock outbreaks.

Herd No.	Host	State	County	Lineage	Year Detected	MRCA date (95% HPD)	Time to detection (years)	PP
1	Cattle*	WY	Fremont	L5	1989	1988 (1985-1989)	1	1.00
2	Cattle	ID	Fremont	L5	2002	1996 (1991-1999)	6	1.00
3	Cattle	WY	Teton	L1	2004	2003 (2001-2004)	1	0.14
4	Cattle	WY	Sublette	L3	2004	1992 (1986-1997)	12	1.00
5	Cattle	ID	Bonneville	L4	2005	1997 (1990-2002)	8	1.00
6	Cattle*	WY	Sublette	L1	2008	2006 (2004-2008)	2	0.35
7	Cattle†	ID	Jefferson	L4	2009	1998 (1991-2004)	11	1.00
8	Bison	MT	Gallatin	L5	2010	2007 (2003-2010)	3	1.00
9	Cattle	WY	Park	L5	2010	2010 (2008-2010)	0	1.00
10	Bison‡	WY	Park	L5	2010	2009 (2007-2010)	1	1.00
11	Cattle	MT	Park	L2	2011	2010 (2008-2011)	1	1.00
12	Cattle	WY	Park	L5	2011	2009 (2006-2011)	2	1.00
13	Cattle	WY	Park	L5	2011	2011 (2010-2011)	0	1.00
14	Bison	ID	Bonneville	L1	2012	2011 (2010-2012)	1	1.00
15	Cattle†	ID	Fremont	L4	2012	2011 (2010-2012)	1	1.00

MRCA, Most recent common ancestor; 95% HPD, 95% Highest probability density interval; PP, posterior probability

*Elk isolate nested within livestock cluster

†Genetically-linked cattle outbreaks: 2009 outbreak in Jefferson County, ID ancestral to 2012 outbreak in Fremont County, ID.

‡Single isolate from herd fell outside group, clustering with elk. Possible independent transmission event, and was not included in MRCA date estimate.

Supplementary Table 7. Model estimates for host state transitioning between pairs of hosts when considering four host states: elk, wild bison (w), domestic bison (d) and cattle.

FROM	TO	Rate (95% HPD)*	# Jumps (95% HPD) †	BF‡
Elk	*Bison (w)*	0.75 (0.03-1.78)	5.02 (1.97-8.31)	84
	Bison (d)	0.78 (0.10-1.71)	5.22 (4.93-5.80)	80978
	Cattle	1.53 (0.29-3.11)	10.66 (8.95-13.44)	80978
Bison (wild)	*Elk*	2.56 (0.58-5.04)	12.97 (7.27-20.31)	80978
	Bison (d)	0.10 (0.00-2.86)	0.00 (0.00-0.05)	0
	Cattle	0.34 (0.00-2.35)	0.00 (0.00-1.98)	0
Bison (domestic)	*Elk*	0.28 (0.00-2.61)	0.00 (0.00-0.24)	0
	Bison (w)	0.28 (0.00-2.57)	0.00 (0.00-0.16)	0
	Cattle	0.24 (0.00-2.73)	0.00 (0.00-0.12)	0
Cattle	*Elk*	0.38 (0.00-2.43)	0.00 (0.00-2.43)	0
	Bison (w)	0.32 (0.00-2.34)	0.00 (0.00-0.24)	0
	Bison (d)	0.21 (0.00-2.71)	0.00 (0.00-0.04)	0

*Rate represents the mean number of host transitions per year. 95% HPD, 95% highest probability density interval

†Median estimate of the total number of Markov jumps over the phylogeny

‡Bayes Factor (BF) support values
